# Clinical Diagnostic Criteria Have a High Impact on the Frequency of Dementia in Late-Stage Parkinson's Disease

**DOI:** 10.3389/fneur.2021.652424

**Published:** 2021-05-20

**Authors:** Catarina Severiano e Sousa, Margherita Fabbri, Catarina Godinho, Rita Simões, Inês Chendo, Miguel Coelho, Isabel Pavão Martins, Joaquim J. Ferreira

**Affiliations:** ^1^Laboratory of Clinical Pharmacology and Therapeutics, Faculdade de Medicina, Universidade de Lisboa, Lisbon, Portugal; ^2^Instituto de Medicina Molecular João Lobo Antunes, Faculdade de Medicina, Universidade de Lisboa, Lisbon, Portugal; ^3^Department of Neurosciences, Clinical Investigation Center 1436, Parkinson Toulouse Expert Center, NS-Park/FCRIN Network and NeuroToul COEN Center, Toulouse University Hospital, INSERM, University of Toulouse 3, Toulouse, France; ^4^Grupo de Patologia Médica, Nutrição e Exercício Clínico do Centro de Investigação Interdisciplinar Egas Moniz, Escola Superior de Saúde Egas Moniz, Almada, Portugal; ^5^Neurology Department, Hospital Beatriz Ângelo, Loures, Portugal; ^6^Campus Neurológico Sénior, Torres Vedras, Portugal; ^7^Psychiatry Department, Department of Neurosciences, Hospital de Santa Maria, Lisbon, Portugal; ^8^Clínica Universitária de Psiquiatria, Faculdade de Medicina, Universidade de Lisboa, Lisbon, Portugal; ^9^Neurology Service, Department of Neurosciences, Hospital Santa Maria, Lisbon, Portugal; ^10^Laboratório de Estudos da Linguagem, Instituto de Medicina Molecular de Lisboa, Faculdade de Medicina, Universidade de Lisboa, Lisbon, Portugal

**Keywords:** Parkinson's disease, late-stage, dementia, cognitive impairment, diagnostic criteria

## Abstract

**Background:** Cognitive impairment is a potential late feature of Parkinson's disease (PD). However, studies in patients with late-stage PD are lacking due to the particular characteristics of this population.

**Objectives:** To evaluate the frequency of dementia in late-stage PD patients and to assess the impact of using different diagnostic criteria.

**Methods:** We conducted a cross-sectional study to estimate the frequency of dementia in late-stage PD patients using the International Parkinson and Movement Disorders Society (MDS) (Level II) clinical diagnostic criteria as the primary outcome. We also applied other diagnostic criteria [MDS (Level I), DSM-IV, and DSM-5 criteria] to determine their applicability and impact on dementia frequency.

**Results:** 93 participants with a mean age of 75.8 years (SD 6.8) and 16.5 years (SD 7.5) of disease duration were included. 64.3% were classified as demented using the International Parkinson and Movement Disorders Society (MDS) (Level II) clinical diagnostic criteria. We observed a high discrepancy on the frequency of dementia depending on the criteria applied [6.2% with MDS (Level I), 58.8% with DSM-IV, and 72.0% with DSM-5 criteria].

**Conclusions:** We found a frequency of dementia below what was observed in similar populations. The particular characteristics of our sample may have contributed as protective factors for late-stage dementia. Dementia frequency varied depending on the criteria used mainly due to the presence of major depression.

## Introduction

Cognitive impairment is a late clinically feature of Parkinson's disease (PD). Dementia is a potential late-stage complication of PD, which may affect multiple cognitive domains (1) causing disability (2) and patient institutionalisation (3–6). However, there is no consensus on dementia frequency partly because the diagnostic criteria that have been used vary from the DSM-IV criteria with brief cognitive assessment (2, 7) to quantitative neuropsychological assessment (8, 9).

The International Parkinson and Movement Disorder Society (MDS) recommends clinical diagnostic criteria for “probable” and “possible” Parkinson's disease dementia (PDD) (10) and specific diagnostic procedures based on a two-level process, depending on the clinical scenario and evaluator expertise (11): Level (I) short algorithm to be used as a screening tool, with a checklist format; Level (II) a detailed neuropsychological assessment (NPA), with proposed cognitive tests that can confirm PDD, characterise its components, and facilitate the differential diagnosis between PDD and other diagnoses.

Although it is already well-accepted that MDS PDD Level II clinical criteria are the gold standard for PDD diagnosis, to our knowledge, there are no published studies on the dementia frequency by using updated PDD diagnostic criteria with late-stage PD patients (LSPD), which is a special population, often understudied due to its particular characteristics. As such, the current study was designed to evaluate the frequency of dementia and determine the applicability and impact of using different diagnostic criteria in LSPD.

## Materials and Methods

### Recruitment

In this cross-sectional study, consecutive LSPD patients attending the Movement Disorders Unit of the University Hospital of Santa Maria (Lisbon) were recruited.

All participants and their formal/informal caregivers were informed about the study objectives and procedures and were asked for their written informed consent. The informed consent of cognitively impaired patients was signed by the caregiver. The study was approved by the local ethics committee.

### Inclusion/Exclusion Criteria

Inclusion criteria were (1) Idiopathic PD according to the UK Brain Bank criteria (12); (2) LSPD (2) [patients who are highly dependent on caregivers for ADL, owing to treatment-resistant motor symptoms or non-motor symptoms, with a Hoehn and Yahr scale (H&Y) score >3 (13) and/or a Schwab and England scale (S&E) score <50% (14) in the MED ON condition] and (3) signed informed consent.

Exclusion criteria were (1) dementia before PD onset and (2) dementia within 1 year following PD diagnosis.

### Outcomes

#### Primary Outcome

The frequency of dementia was determined using MDS clinical criteria for “probable” and “possible” PDD, operationalized according to the Level II procedures outlined by Dubois et al. (11) which were based on neuropsychological tests, functional autonomy as well as clinical interview. Briefly, involved the following: (1) PD; (2) PD before dementia; (3) decreased global cognitive efficiency evidenced by education-adjusted Mini-Mental State Exam [Portuguese cutoff: score <22 (0–2 yrs); <24 (3–6 yrs); <27 (≥7 yrs)]; (4) cognitive deficits that impact activities of daily life (ADL); (5) deficits in two or more cognitive domains documented by a detailed NPA; (6) the presence of at least one behavioural symptom (apathy, depressed mood, hallucinations, delusions, or excessive daytime sleepiness) supports the diagnosis of probable PDD; (7) the presence of any abnormality which may by itself cause significant cognitive impairment (e.g., delirium, relevant vascular disease) but judged not to be the cause of dementia makes the diagnosis uncertain and supports the diagnosis of possible PDD.

#### Secondary Outcomes

Frequency of dementia according to three other diagnostic criteria:

1) MDS PDD Level I (11) − Level I and Level II are broadly the same criteria but Level I has some particularities: (1) the methodology to assess cognition is brief and (2) the absence of major depression, *delirium*, and abnormalities that may obscure PDD diagnosis is mandatory to diagnose probable PDD. In the presence of these abnormalities, dementia is not excluded but we have to perform the Level II NPA to diagnose possible PDD.
2) DSM-IV criteria (15) − Although the DSM-5 is already published, which makes DSM-IV criteria no longer clinically relevant, we considered it important to use them because most of the previous studies used the DSM-IV criteria to diagnose PDD and we intended to perform a comparative analysis of the frequency of dementia with other studies that used similar samples.
3) DSM-5 criteria (16) − Briefly, these criteria required the following steps:
  - Distinction between mild and major Neurocognitive Disorder (NCD). Major NCD corresponds to the diagnosis of dementia. Its main feature is the presence of a significant acquired cognitive decline, which may be documented by:
    - detailed NPA, considered as cognitive impairment performance values below 2 standard deviations (SD) on age-and-educated normative scores in one or more cognitive domains, or
    - brief but quantitative cognitive assessment.
  - Identification of behavioural symptoms that support the diagnosis of PDD.
  - Specification of the level of certainty of dementia diagnosis: major NCD probably due to PD vs. major NCD possibly due to PD. What distinguishes them is the presence of abnormalities that may obscure diagnosis.

We compared dementia frequency obtained through the two DSM-5 possible approaches (NPA vs. brief cognitive evaluation), also testing its applicability in LSPD.

### Measures

#### Neurologic and Functional Assessment

Neurological assessment was performed by a neurologist with expertise in movement disorders and included the investigation of vascular problems. Disease severity and disability were assessed using H&Y (13) and S&E (14) scales, respectively.

#### Neuropsychiatric and Behavioural Assessment

The neuropsychiatric assessment was performed by the neurologist and included neuropsychiatric functions (behavioural symptoms and major depression). The neuropsychiatric inventory (17) (NPI) and the geriatric depression scale (18) (GDS) were used, respectively.

To diagnose *delirium*, we used the DSM-5 criteria (16) as the MDS criteria (11) omit the methodology that we were to use.

#### Neuropsychological Assessment

The NPA was performed by a neuropsychologist. It took place in a single session, at the patients' homes, in order to ensure the presence of the usual amenities, and to reduce as far as possible any interference, malaise, or interfering in the patients' lives.

A clinical interview was undertaken with caregiver collaboration to obtain the following information: (1) demographics; (2) educational history; (3) temporal relationship of PD diagnosis and the onset of cognitive complaints; (4) disease duration; (5) objective and subjective cognitive complaints; (6) pattern of PD and cognitive complaints progression; (7) impact of cognitive impairment on ADL; (8) history of abnormalities that may obscure the diagnosis (e.g., delirium, vascular or other neurological diseases); (9) current comorbidities.

To assess the impact of cognitive impairment on ADL, two approaches were taken: (1) patient and caregiver clinical interview, using a checklist with cognitive ADL tasks and, (2) the Pill Questionnaire, with the assumption that if the patient was able to manage his/her treatment in the past, the inability to describe one's own medication regimen was equivalent to an impairment in ADL (11). Whenever one of these sources of information was suggestive of dysfunction, the patient was classified as having impaired ADL.

To assess cognition using MDS PDD Level II criteria (11), we proceeded with NPA with a set of cognitive tests selected from their proposal (Table 1), according to the following criteria: (1) existence of normative data for the Portuguese population, (2) tests predictably less sensitive to PD manifestations, and (3) expected time consumed by the test (only one test for each cognitive task whose performance would have a shorter estimated duration, to ensure the minimum tiredness interference). We considered cognitive impairment performance values below 1 SD on age-and-educated-adjusted normative scores in two or more cognitive domains. We applied neuropsychological tests in the same order for all patients.

**Table 1:** Neuropsychological tests used to assess cognition according to MDS PDD Level I and Level II criteria.

**Cognitive domains/tasks**	**MDS PDD Level I cognitive tests**	**MDS PDD Level II cognitive tests**
**Global efficiency**	MMSE (19, 20)	MMSE (19–22)
**Attention**	Serial 3's of the MMSE (19)	
**Executive functions**	Phonologic fluency (P) (23, 24)	
Working memory		Digit span (25, 26)
Conceptualisation		Similarities (WAIS-III) (27, 28)
Set activation		Phonologic fluency (P, M, R) (23, 24)
Set shifting		TMT (A and B) (29, 30)
Set maintenance		Odd man out test (31)
Behavioural control		Prehension behaviour (FAB) (32, 33)
**Memory**	3-Word Recall of the MMSE (19)	RAVLT (34, 35)
**Instrumental functions**		
Language		Boston naming test (36)
Visuoconstructive	Drawing of the MMSE Pentagons (19)	Copy of the clock (37)
Visuospatial		Benton line orientation test (38)
Visuoperceptive		Benton face recognition test (39)

*MDS PDD, Parkinson's Disease dementia criteria recommended by Movement Disorder Society Task Force (11); MMSE, Mini-Mental State Exam; TMT (A and B), Trail Making Test A and B; WAIS-III, Wechsler Adult Intelligence Scale 3rd Edition; FAB, Frontal Assessment Battery; RAVLT, Rey Auditory and Verbal Learning Test*.

To assess cognition following MDS PDD Level I criteria (11), we used the proposed cognitive tests (Table 1), adapted to the Portuguese population.

To assess cognition according to the DSM-IV criteria (15), we used MMSE sub-scales (19) to assess memory, aphasia, agnosia and apraxia (retention and evocation, language and construction sub-scales, respectively). To assess executive function, we used WAIS-III similarities sub-scale (28), MMSE attention and calculation sub-scale (19), phonologic fluency test (P, M, R letters) (24) and information collected in the clinical interview (impact of cognitive impairment on ADL such as the ability to plan daily activities).

For DSM-5 detailed NPA (16), we used the same tests selected from MDS PDD Level II (11) but we strictly followed the DSM-5 proposed cognitive domains organisation and cognitive impairment definition. As social cognition is a cognitive domain that is not included in the proposed MDS criteria, we followed the DSM-5 assessment methodology and, to guarantee the non-interference of poor insight, we assessed it during the clinical interview with the contribution of the caregiver. As the evaluation of this domain was qualitative, we considered as a reference for impairment the examples of major symptoms made available by the DSM-5.

For brief DSM-5 cognitive assessment (16), we used the MMSE (19) even though it is less effective in assessing global PD cognition. This decision was mainly due to two reasons: (1) the MMSE is commonly used due to its quick and ease of use and access and (2) the MMSE was proposed by the MDS PDD criteria (11) as a measure of global cognitive efficiency. As it is validated for the Portuguese population and there are several cutoff proposals, we decided to perform a comparative analysis with the cutoff that is the most used in clinical practise [adjusted for education (20), age and education (21), dementia diagnosis (22)].

### Statistical Methods

#### Sample Size

Assuming an expected LSPD dementia frequency of ~50% (2), with a 95% confidence interval and an error level of ~10%, a sample of 100 patients was estimated necessary.

#### Statistical Analysis

Statistical analysis was performed using SPSS software version 26 (IBM SPSS, Chicago, IL).

We used descriptive statistics to characterise demographic and clinical information. Categorical variables were described through absolute and relative frequencies (%) and continuous variables through the mean value and standard deviation (SD).

The frequency of dementia was analysed using descriptive statistics. We presented the absolute and relative frequency (%) of LSPD dementia cases, according to the different criteria for dementia that we used.

The agreement analysis between our gold-standard and the other criteria for PDD was performed using Cohen's Kappa (>0.6 is significant) (40). We calculated sensitivity, specificity, positive predictive value, and negative predictive value for each PDD diagnostic criteria.

We considered a confidence interval of 95%.

## Results

### Patient Demographics and Clinical Characteristics

**Figure 1:**
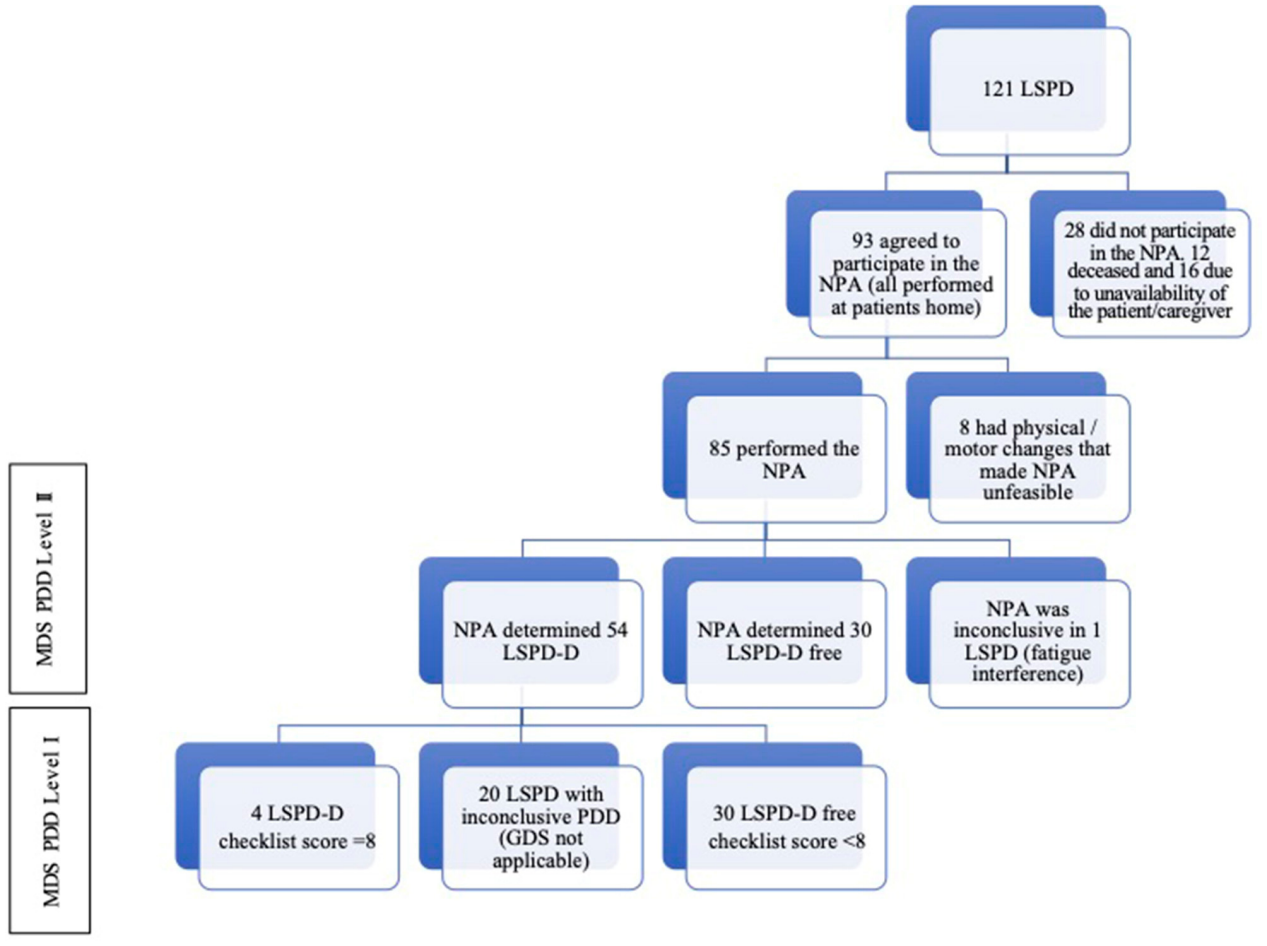
Patients and assessments flowchart. MDS PDD, Parkinson's Disease dementia criteria recommended by Movement Disorder Society Task Force (11); LSPD, Late-stage Parkinson's Disease patients; LSPD-D, Late-stage Parkinson's Disease dementia patients; NPA, Neuropsychological assessment; GDS, Geriatric Depression Scale.

One hundred and twenty-one LSPD patients were initially recruited (Figure 1). Following motor assessment but before the proposed NPA, 12 patients died and 16 declined to participate in the NPA (13 patients refused to perform it and three were unable to due to the unavailability of the caregiver). A total of 93 LSPD patients agreed to perform the NPA. However, when patients were visited at home, the neuropsychologist found that it would not be possible to carry out the NPA of eight patients due to their physical/motor disability. Of these eight patients, we only collected information through the neurological and neuropsychiatric evaluation, and through the clinical history that we performed with the help of the caregiver (Table 2).

**Table 2:** Demographic and clinical characteristics of LSPD patients.

	**LSPD (*N* = 85) Mean (SD)**	LSPD (*N* = 8)^*^ Mean (SD)	**LSPD (*N* = 93)** ** Mean (SD)**
Gender (m/f)	36/49	4/4	40/53
Age (yrs)	75.4 (6.9)	80.3 (4.7)	75.8 (6.8)
Education (yrs)	6.5 (4.5)	3.1 (1.6)	6.4 (4.6)
Disease duration (yrs)	16.9 (7.5)	12.0 (6.5)	16.5 (7.5)
Age at onset (yrs)	58.5 (10.9)	66.3 (4.6)	59.1 (10.8)
Hoehn and Yahr stage (0/5)	4.1 (0.9)	5 (0)	4.2 (0.9)
Schwab and England (0/100%)	36.7 (13.6)	15.71 (7.9)	35.1 (14.3)
NPI delusions (0/12) (Cutoff ≥3)	1.3 (2.6)	0.3 (0.8)	1.3 (2.5)
NPI hallucinations (0/12) (Cutoff ≥3)	1.7 (2.7)	1.7 (1.9)	1.7 (2.7)
NPI depression (0/12) (Cutoff ≥3)	3.3 (2.4)	1.8 (2.0)	3.2 (2.4)
NPI apathy (0/12) (Cutoff ≥3)	3.6 (3.6)	3.8 (4.3)	3.6 (3.6)
NPI sleep disorders (0/12) (Cutoff ≥3)	2.5 (3.3)	1.0 (1.5)	2.4 (3.3)
Pill Questionnaire (0/3) (Cutoff ≥2)	2.2 (1.1)	3 (0)	2.3 (1.0)
GDS Score (0/30) (Cutoff 11–20: mild depression; 21–30: severe depression)	13.8 (6.8)	NA	NA
MMSE Score (0/30) (Cutoff: 0–2 yrs education-22 pt; 3–6 yrs education- 24 pt; ≥7 yrs education-27 pt)	21.6 (6.1)	NA	NA

*LSPD, Late-stage Parkinson's Disease; NPI, Neuropsychiatric Inventory; GDS, Geriatric Depression Scale; MMSE, Mini-Mental State Exam; NA, Not applicable due to physical/motor impairment*.

* *These 8 patients did not perform the neuropsychological assessment due to the physical and motor limitations they presented. This information was collected through the caregiver-based clinical interview that we performed*.

In total, 85 patients underwent complete NPA (Figure 1). Forty-nine (57.6%) were women, with a mean age of 75.4 years (SD 6.9), 6.5 years (SD 4.5) of education, 16.9 years (SD 7.5) of disease duration, age at PD onset (58.5 years; SD 10.9), a mean H&Y of 4.1 (SD 0.9) and loss of independence assessed by the S&E corresponding to 36.7% (SD 13.6) (Table 2).

### Dementia Frequency According to MDS PDD Level II Criteria

Fifty-four (64.3%) patients met the criteria for PDD [27/36 men (75%), 27/49 women (55%)]: 47 (55.9%) were diagnosed with probable PDD and seven (8.3%) with possible PDD due to the abnormalities that made de diagnostic uncertain (3 LSPD had a stroke significantly before PD, three were submitted to undergo deep brain stimulation after PD and one had an oncological disease and was undergoing chemotherapy after the beginning of cognitive impairment). According to the caregiver, none of these abnormalities was associated with the onset of worsening cognitive impairment. One patient had inconclusive PDD due to the interference of tiredness during NPA.

69.4% LSPD presented an MMSE score lower than the education cutoff. The mean score obtained was 21.6 (SD 6.1), with an interval ranging from eight to 30 points. 67.9% of LSPD had impaired cognition in two or more cognitive domains and 87.1% presented cognitive changes severe enough to impair ADL.

We observed at least one behavioural symptom with significant expression in 74.7% of LSPD (Table 3). The most common was depressive mood (57.8%), followed by apathy (51.8%), excessive daytime sleepiness (36.1%), hallucinations (25.3%), and delusions (18.1%).

**Table 3:** Diagnostic rating sheet for Probable PDD recommended by the MDS Task Force.

**LSPD (*N* = 85)**	**MDS PDD Level I**	**MDS PDD Level II**
	Yes (%)	Yes (%)
1.Parkinson's disease	100	100
2.Parkinson's disease developed before dementia	100	100
3.MMSE^**^ <22 (0–2 yrs education); <24 (3–6 yrs education); <27 (≥7 yrs education)	69.4	69.4
4.Dementia has impact on ADLs	87.1	87.1
5.Impaired cognition (for yes, at least of 2 of 4 cognitive domains below are abnormal)	79.1	67.9
5 a) Attention	48.2	^*^
5 b) Executive Function	87.1	56.5
B1) Working memory	^*^	25.9
B2) Conceptualization	^*^	11.8
B3) Set activation	^*^	68.7
B4) Set shifting	^*^	85.9
B5) Set maintenance	^*^	55.2
B6) Behavioral control	^*^	30.1
5 c) Instrumental Functions	^*^	55.6
C1) Language	^*^	11.1
C2) Visuo-Constructive	65.5	60.8
C3) Visuo-Spatial	^*^	67.1
C4) Visuo-Perceptive	^*^	61.1
5 d) Memory	62.4	68.7
D1) Only medial temporal/hippocampal component	^*^	18.1
D2) Only subcortico-frontal component	^*^	15.7
D3) Both components	^*^	44.6
6.Absence of major depression	18.6	^*^
7.Absence of *delirium*	98.8	^*^
8.Absence of other abnormalities that obscure diagnosis	89.4	89.4
Behavioral symptoms (one of delusions, hallucinations, depression, excessive daytime sleepiness)	74.7	74.7
Probable PDD (items 1–8 must all be YES)	6.2	55.9^***^
Probable PDD Inconclusive	23.5	1.2
Possible PDD	0	8.3
Total Score (0/8) Mean (SD)	6.3 (1.0)	^*^

*MDS PDD, Parkinson's Disease dementia criteria recommended by Movement Disorder Society Task Force (11); PDD, Parkinson's Disease dementia; MMSE, Mini-Mental State Exam; ADLs, Activities of daily living.*

* *Not applicable in this Level.*

** *We considered education-adjusted MMSE cutoff scores (Portuguese population)*.

*** *Items 6 and 7 not applicable for MDS PDD Level II*.

Of the eight patients who could not undergo NPA, it was possible to confirm through the clinical history collected from the caregivers that before the worsening of the physical/motor impairment significant global cognitive impairment with impact on ADL had occurred, leading us to suspect the existence of PDD. They were older patients, less educated, and who, despite having fewer years of PD, had worse disease severity and dysfunction (Table 2).

If we were to consider them as having PDD, we would have observed a frequency of dementia of 67.4% (Table 4).

**Table 4:** Dementia frequency according to different diagnostic criteria.

**Diagnostic criteria**	**LSPD (*N* = 85) (%)**	LSPD (*N* = 93)^*^ (%)
MDS PDD Level I	6.2	16.4
Inconclusive (major depression not assessed - difficulty GDS items comprehension)	23.5	
MDS PDD Level II	64.3	67.4
**Probable** PDD	55.9	
**Possible** PDD	8.3	
Inconclusive (fatigue interference did not allow to confirm PDD diagnosis)	1.2	
DSM-IV	58.8	62.4
DSM-5		
With MMSE adjusted for education	62.4	65.6
Major NCD **Probably** due to PD **with** behavioural disturbance	48.2	
Major NCD **Probably** due to PD **without** behavioural disturbance	7.1	
Major NCD **Possibly** due to PD	7.1	
With MMSE adjusted for age and education	68.2	70.9
Major NCD **Probably** due to PD **with** behavioural disturbance	52.9	
Major NCD **Probably** due to PD **without** behavioural disturbance	8.2	
Major NCD **Possibly** due to PD	7.1	
With MMSE adjusted for dementia	60.0	63.4
Major NCD **Probably** due to PD **with** behavioural disturbance	47.1	
Major NCD **Probably** due to PD **without** behavioural disturbance	5.9	
Major NCD **Possibly** due to PD	7.1	
With NPA	72.0	74.4
Major NCD **Probably** due to PD **with** behavioural disturbance	56.1	
Major NCD **Probably** due to PD **without** behavioural disturbance	7.3	
Major NCD **Possibly** due to PD	8.5	
Inconclusive (fatigue interference did not allow differential diagnosis between MCI and PDD)	3.5	

*LSPD, Late-stage Parkinson's Disease patients; PDD, Parkinson's Disease dementia; MDS PDD, Parkinson's Disease dementia criteria recommended by Movement Disorder Society Task Force (11); DSM-IV, Parkinson's Disease dementia criteria recommended by Diagnostic and Statistical Manual of Mental Disorders – Fourth Edition (15); DSM-5, Parkinson's Disease dementia criteria recommended by Diagnostic and Statistical Manual of Mental Disorders – Fifth Edition (16); NCD, Neurocognitive disorder; GDS, Geriatric Depression Scale; MMSE- Mini-Mental State Exam; NPA, Neuropsychological assessment; MCI, Mild cognitive impairment*.

* *If we considered as PDD the 8 LSPD who could not perform the NPA due to physical/motor disabilities, based on the clinical history collected from the caregiver*.

### Dementia Frequency According to Three Other PDD Diagnostic Criteria

The frequency of dementia differed markedly according to the diagnostic criteria used. Dementia frequency rated by MDS Level I was consistently the lowest. Only 6.2% of the LSPD met all screening checklist clinical criteria to be diagnosed as probable PDD (Table 3). All patients identified by the screening checklist as having PDD were confirmed by Level II criteria (100% specificity). However, Level II criteria identified more patients with PDD than the screening checklist (sensitivity 8.8%) (Table 5). Although 79.1% of LSPD presented impaired cognition in two or more cognitive domains and 87.1% of LSPD presented cognitive changes severe enough to impair ADL, we observed the absence of major depression that is mandatory for Level I PDD diagnosis only in 18.6% of LSPD. In the same way, 23.5% of LSPD had Level I PDD inconclusive due to comprehension difficulties that made GDS not applicable and impossible to conclude about the absence of major depression.

**Table 5:** Classification of LSPD by different diagnostic criteria using MDS PDD Level II criteria as the gold-standard.

**Classification**	**MDS PDD Level I**	**DSM-IV**	**DSM-5 MMSE adjusted for education**	**DSM-5** ** MMSE adjusted for age and education**	**DSM-5 MMSE adjusted for dementia**	**DSM-5 NPA**
True positive	3	45	52	54	49	54
False positive	0	5	0	3	2	5
True negative	30	25	30	27	28	23
False negative	31	9	2	0	5	0
PPV (%)	100	90.0	100	94.7	96.1	91.5
NPV (%)	49.2	73.5	93.8	100	84.8	100
Sensitivity (%)	8.8	83.3	96.3	100	90.7	100
Specificity (%)	100	83.3	100	90.0	93.3	82.1
Kappa coefficient^*^	0.1	0.6	0.9	0.9	0.8	0.9

*LSPD, Late-stage Parkinson's Disease patients; MDS PDD, Parkinson's Disease dementia criteria recommended by Movement Disorder Society Task Force (11); DSM-IV, Parkinson's Disease dementia criteria recommended by Diagnostic and Statistical Manual of Mental Disorders – Fourth Edition (15); DSM-5, Parkinson's Disease dementia criteria recommended by Diagnostic and Statistical Manual of Mental Disorders – Fifth Edition (16); MMSE, Mini-Mental State Exam; NPA, Neuropsychological assessment; PPV, Positive Predictive Value; NPV, Negative Predictive Value*.

* *Kappa coefficient >0.6 is significant*.

Although less markedly, we also observed discrepant results in dementia frequency between DSM-IV diagnostic criteria (58.8%) and Level II criteria (64.3%).

Using DSM-5 with full NPA criteria, once again, we observed discrepant results (72.0 vs. 64.3%). Three patients (3.5%) were given an inconclusive dementia diagnosis due to the interference of tiredness which did not allow differential diagnosis between Mild Cognitive Impairment and PDD (Table 4).

When we used the DSM-5 criteria with MMSE, we observed results quite similar to Level II criteria (62.4% with education adjustment, 68.2% with age and education adjustment, and 60.0% with dementia adjustment vs. 64.3%, respectively).

When we analysed the agreement between Level II and DSM-5 criteria (Table 5), we observed the significant similarity seen in clinical practise. The DSM-5 was very sensitive in identifying demented LSPD, either through the use of the NPA (100%) or through the use of MMSE (adjusted for education 96.3%; age and education 100%; diagnosis of dementia 90.7%).

The specificity of the DSM-5 with MMSE adjusted for age and education (90.0%) and with NPA (82.1%) was lower than the specificity obtained with MMSE adjusted only for education and for the diagnosis of dementia because the first diagnosed more dementia than the MDS Level II criteria, so they presented a higher number of false positives.

## Discussion

Our results conclude that by using MDS Level II (11) clinical diagnosis criteria, slightly more than half of the 85 LSPD (64.3%) meet dementia criteria. Considering the high frequency of dementia (80%) reported by Reid et al. (9) who studied a sample of PD with 20 years of disease, we expected to find similar results. When we compared the frequency of dementia that we obtained through the same diagnostic criteria used by Reid et al. (9), we observed a greater discrepancy (58.8 vs. 80%). We hypothesised that this discrepancy could be due in part to the fact that Reid et al. (9) diagnosed PDD using the DSM-IV criteria with full NPA to assess cognition, contrary to what we did in our study where we strictly followed the DSM-IV criteria using a brief cognitive assessment methodology mainly with MMSE sub-scales, which, as is well-known, is not the best methodology for assessing cognition in PD. However, our analysis of agreement between diagnosis criteria allowed us to conclude that the MMSE proved to be a reliable tool to diagnose dementia in LSPD. The discrepancy that we observed may have been due in part to the clinical and socio-demographic characteristics of our sample that should be noted.

Our participants had been living with PD for an average of 17 years, with an early age at PD onset (<60 years) and there was a higher proportion of women (58%) than expected based on gender prevalence of PD. Forsaa et al. (4) developed a long-term study about mortality and associated risk factors in a population-based cohort with PD and found that approximately 70% of patients with a median survival from motor onset of 16 and 20 years after disease onset had died. They concluded that higher age at onset, male sex, and dementia were independent risk factors for mortality. Cholerton et al. (41) compared baseline cognitive, demographic, and clinical characteristics of participants who remained cognitively stable and those who progressed throughout follow-up in a large, well-characterised PD cohort and found that the primary predictive factor in the transition from no cognitive impairment to cognitive impairment was male sex. They also found that female participants progressed more slowly to cognitive impairment than male participants. We believe that ~64% of PDD we have identified through our gold standard may be due to this conjuncture of characteristics, namely the fact that about 58% of the participants were female. Of the 36 (42.4%) men who participated, 27 (75%) were diagnosed with PDD and of the 49 (57.6%) participating women, 27 (55%) obtained the same diagnosis, which corroborates the results found by Cholerton et al. (41) and may justify the fact that our population was mostly female. This may be another peculiarity of LSPD. It can be hypothesised that female gender may be a protective factor.

On the other hand, the participants had a level of education (mean of 6 years) that is not representative of the aged Portuguese population who is poorly educated. According to the National Institute of Statistics (Source: PORDATA), in 2019 only about 4.6% of the general population over 65 years had 6 years of schooling. The remaining 73% had no education or only up to 4 years of schooling and about 22.5% had nine or more years of schooling. Considering that the results of cognitive tests were corrected in general for age and education based on the mean values obtained for the general aged Portuguese population, education may also have contributed to the lower frequency of dementia found.

As we expected, the frequency of dementia was partly dependent on the diagnostic criteria used. We found a significant discrepancy between the results obtained with the MDS PDD Level I and Level II criteria (11) (6.2 vs. 64.3%, respectively). The checklist proposed at Level I proved to be quite specific in identifying non-demented LSPD but very unresponsive in identifying demented LSPD. The agreement between the two diagnostic criteria was not significant. The mandatory absence of major depression was largely responsible for different dementia frequency and for the lack of significant agreement among the two levels. Only 18.6% of the LSPD was not diagnosed with major depression and 23.5% had an inconclusive diagnosis due to their difficulty in understanding the GDS (18) items. These results are concordant with the results published by Barton et al. (42) who identified similar results.

When we used the DSM-5 criteria (16), we observed less discrepancy. The agreement analysis demonstrate a significant concordance between DSM-5 and Level II criteria. However, with the NPA, there was an increase in dementia frequency (72.0 vs. 64.3%, respectively). The lowest number of impaired cognitive domains that DSM-5 criteria considers sufficient to diagnose dementia justified it.

When we used DSM-5 criteria (16) with the MMSE, the frequency of dementia was quite similar (62.4% with education adjustment, 68.2% with age and education adjustment, 60.0% with dementia adjustment). These results revealed that MMSE assessed cognitive domains (like orientation and complex orders comprehension) that were not included in NPA and proved important in the cognitive profile of these patients, which is more similar to normal ageing, with global impairment.

In terms of clinical translation and practical application, the good specificity of Level I criteria proved that this is a time-efficient method to exclude PDD. However, as the absence of major depression is mandatory to perform the Level I diagnosis, future studies in similar populations will be important to confirm whether major depression is a frequent co-morbidity and characteristic of late-stage PD, as this influences how we should evaluate it and to conclude about the applicability of MDS Level I criteria in this population. Such an evaluation should likely be performed based on a semi-structured clinical interview.

On the other hand, DSM-5 criteria proved to be more efficient to diagnose dementia, with a high positive predictive value and good specificity in identifying non-demented patients. However, due to the false positives that may be diagnosed with these criteria, conclusions about dementia diagnosis must be cautious and well-supported by a robust clinical interview with the caregiver collaboration to assess the clinical manifestations and the impact of cognitive impairment in ADL.

None of the criteria used to diagnose dementia applied to the eight patients with severe physical/motor disabilities for whom NPA was impossible. In the future, we consider it important that caregiver-based qualitative assessment methodology be developed to make conclusions on PDD in patients with these typical late-stage physical/motor disabilities, otherwise, we risk underestimating it.

The strengths of our study were the high number of LSPD patients who participated in the NPA and were assessed at home. Considering their disease severity, we believe it was a huge effort and willingness to collaborate in our study.

Given the particular clinical and demographic characteristics of our sample, we believe that our results may be compared with future studies with similar populations to inform about the presence of eventual protective factors associated with the late-stage PD dementia.

This study has also some limitations that we must consider. Firstly, given that the present data come from outpatients attending a specialist movement disorder unit, the results need to be validated in a community sample. Secondly, we used a cross-sectional design and, to confirm the validity of PDD diagnosis procedures, it would be of interest to have a prospective cohort and longitudinal data. Finally, the inexistence of a no-late-stage control group (e.g., advanced stage) didn't allow us to validate the cognitive assessment methodology that we used and analyze the frequency of dementia in a different stage of PD.

In conclusion, our study indicates that below what we would expect, slightly more than half the participants meet dementia criteria. This must have been due to the particular characteristics of our LSPD sample (early age at PD onset, female sex, higher educational level in relation to the general aged Portuguese population), which may have been protective factors for late-stage PD dementia. The estimates of late-stage dementia frequency were partly dependent on the diagnostic criteria. When screening for PDD in LSPD it is important to consider the possibility of using a caregiver-based qualitative methodology assessment to make conclusions on patients with severe physical/motor disabilities for whom neuropsychological tools cannot be used.

## Data Availability Statement

The raw data supporting the conclusions of this article will be made available by the authors, without undue reservation.

## Ethics Statement

The studies involving human participants were reviewed and approved by Centro Académico de Medicina de Lisboa (Centro Hospitalar Lisboa Norte/Faculdade de Medicina de Lisboa/Instituto de Medicina Molecular João Lobo Antunes). The patients/participants provided their written informed consent to participate in this study.

## Author Contributions

CS: conception, organisation, and execution of the research project, design and execution of the statistical analysis, and writing of the first draught and review and critique of the manuscript preparation. MF, CG, and IC: execution of the research project and review and critique of the manuscript preparation. RS: execution of the research project, review and critique of the statistical analysis, and review and critique of the manuscript preparation. MC: organisation of the research project and review and critique of the manuscript preparation. IM: conceptiona and organisation of the research project, design of the statistical analysis, and review and critique of the manuscript preparation. JF: conception and organisation of the research project, review and critique of the statistical analysis, and review and critique of the manuscript preparation. All authors contributed to the article and approved the submitted version.

## Conflict of Interest

The authors declare that the research was conducted in the absence of any commercial or financial relationships that could be construed as a potential conflict of interest.
